# Emerging Role of Lymphatics in the Regulation of Intestinal Lipid Mobilization

**DOI:** 10.3389/fphys.2019.01604

**Published:** 2020-01-29

**Authors:** Changting Xiao, Priska Stahel, Avital Nahmias, Gary F. Lewis

**Affiliations:** Department of Medicine and Department of Physiology, Banting and Best Diabetes Centre, University of Toronto, Toronto, ON, Canada

**Keywords:** lymphatic, triglyceride, intestine, mobilization, glucose, glucagon-like peptide-2

## Abstract

Intestinal handling of dietary triglycerides has important implications for health and disease. Following digestion in the intestinal lumen, absorption, and re-esterification of fatty acids and monoacylglycerols in intestinal enterocytes, triglycerides are packaged into lipoprotein particles (chylomicrons) for secretion or into cytoplasmic lipid droplets for transient or more prolonged storage. Despite the recognition of prolonged retention of triglycerides in the post-absorptive phase and subsequent release from the intestine in chylomicron particles, the underlying regulatory mechanisms remain poorly understood. Chylomicron secretion involves multiple steps, including intracellular assembly and post-assembly transport through cellular organelles, the lamina propria, and the mesenteric lymphatics before being released into the circulation. Contrary to the long-held view that the intestinal lymphatic vasculature acts mainly as a passive conduit, it is increasingly recognized to play an active and regulatory role in the rate of chylomicron release into the circulation. Here, we review the latest advances in understanding the role of lymphatics in intestinal lipid handling and chylomicron secretion. We highlight emerging evidence that oral glucose and the gut hormone glucagon-like peptide-2 mobilize retained enteral lipid by differing mechanisms to promote the secretion of chylomicrons via glucose possibly by mobilizing cytoplasmic lipid droplets and via glucagon-like peptide-2 possibly by targeting post-enterocyte secretory mechanisms. We discuss other potential regulatory factors that are the focus of ongoing and future research. Regulation of lymphatic pumping and function is emerging as an area of great interest in our understanding of the integrated absorption of dietary fat and chylomicron secretion and potential implications for whole-body metabolic health.

## Introduction

Intestinal handling of dietary triglycerides (TGs) plays an important role in maintaining whole-body lipid and energy homeostasis and has important implications for health and disease (Abumrad and Davidson, 2012). Improved understanding of intestinal lipid processing may help develop novel therapeutic strategies to treat hyperlipidemia and improve cardiac and metabolic health (Lewis et al., 2015). Dietary fat absorption and transport in the gut through lipoprotein secretion is regulated by many factors. Numerous studies have investigated the regulatory mechanisms of intestinal lipoprotein particle biosynthesis and assembly (Xiao et al., 2014; Dash et al., 2015). Recently, it is becoming increasingly appreciated that, besides particle production, regulation of post-assembly transport of lipoproteins contributes to the overall lipid delivery through the gut. While the underlying mechanisms remain incompletely understood, intestinal lymphatics are emerging as a potentially important player. This review aims to examine the interplay between lipoprotein secretion and the function of the mesenteric lymphatic system.

## Dietary Tg Digestion, Absorption, and Chylomicron Synthesis and Secretion

The digestion products of ingested TG, fatty acids and monoacylglycerols, are absorbed at the apical membrane of enterocytes lining the brush border of the small intestinal lumen. Following absorption by enterocytes, fatty acids and monoacylglycerols are re-esterified into TG and form lipid droplets at the ER membrane leaflet. The majority of newly synthesized TGs are packaged into chylomicrons (CMs) for secretion. Lipid droplets bud off into the ER lumen and fuse with a lipid-poor apolipoprotein B48-containing particle to form the pre-chylomicron particle. Pre-chylomicrons are transported in pre-chylomicron transport vesicles (PCTVs) from the ER to the Golgi apparatus where they are further processed. Mature CMs exit enterocytes by exocytosis at the basolateral membrane, pass through the villus lamina propria, enter into the lacteals (a single, blind-ended lymphatic vessel located at the center of each intestinal villus), and move through the mesenteric lymph ducts and then the thoracic duct where they enter the blood circulation at the left subclavian vein.

A portion of lipid droplets are not immediately utilized for pre-chylomicron synthesis. Instead, they bud off the ER into the cytosol, where TGs are utilized for the formation of cytoplasmic lipid droplets (CLDs). CLD synthesis, metabolism, and catabolism are highly dynamic during the fasting and feeding cycle (D’Aquila et al., 2016). CLDs expand in size following a fat meal via either fusion or TG synthesis at the CLD surface, while CLD catabolism during the post-absorptive state occurs through TG lipolysis and redistribution or lipophagy. CLDs may serve as transient storage of TG in enterocytes, which attenuates postprandial TG excursion and provides a continuous TG supply as substrates for late postprandial and post-absorptive CM secretion (Beilstein et al., 2015), enhances the overall efficiency of dietary fat absorption, and prevents cellular and systemic toxicity as a result of lipid overload (Xiao et al., 2018a). It is noted that temporary lipid storage in CLDs is conserved and common in lower species, such as *Caenorhabditis elegans* and reptiles, implicating evolutionary benefits. Dynamic formation and mobilization of CLD lipids are highly regulated and play an important role in determining net CM secretion rate from the intestine. The details of both CM assembly and CLD metabolism have been reviewed elsewhere (Hussain, 2014; Beilstein et al., 2015; D’Aquila et al., 2016). Besides CM secretion and storage in CLDs, dietary fatty acids are also oxidized in enterocytes to generate ketone bodies (Uchida et al., 2011; Schober et al., 2013), a capacity that is well-developed during the suckling period in rodents, disappears at weaning, and can be re-established upon high-fat feeding (Thumelin et al., 1993; Clara et al., 2017; Ramachandran et al., 2018). A high capacity to oxidize dietary fat may translate into a lower risk of weight gain on high fat diets (Kondo et al., 2006). The overall process of dietary fat absorption, CM assembly, and secretion is highly controlled, including regulation of CM biosynthesis (as reviewed Hussain, 2014; Dash et al., 2015) and post-assembly regulation of CM transport prior to entering the circulation (Xiao et al., 2019a).

## Lipid Retention and Mobilization

### Prolonged Post-absorptive Lipid Retention in the Gut

While the majority of dietary TGs are rapidly secreted in CMs during meal ingestion, evidence supports more prolonged retention of TGs in the intestine during the post-absorptive period, with subsequent release triggered by a number of factors (Xiao et al., 2018a, 2019a). In healthy, lean individuals, postprandial plasma TGs rapidly rise, typically peak at 3–4 h after meal, and gradually return to fasting level after 6–8 h. However, under certain circumstances such as ingestion of a second fat meal or glucose, many hours after lipid ingestion, release of TGs from an intestinal “storage” pool can be demonstrated (Xiao et al., 2019a). Indeed, a stable isotope enrichment study in humans demonstrated that TGs in a fat-rich meal appeared in CM up to 18 h after ingestion (Chavez-Jauregui et al., 2010). In humans, abundant lipid droplets are present in the jejunum and duodenum 5 h after fat ingestion (Robertson et al., 2003; Xiao et al., 2018b). In fact, lipid droplets are observed in human duodenum 10 h after high fat ingestion (Xiao et al., 2018b). In rats, lipid deposits are present in the jejunum up to 12 h after fat ingestion (Hung et al., 2017). The physiological significance of such lipid retention in the gut is not clear. It is plausible that it attenuates postprandial excursion of plasma TG. It is also possible that a readily available intestinal lipid pool functions to “prime” the intestinal CM secretory machinery for the next incoming meal. It remains to be established whether such lipid retention in the gut may be abnormal in compromised metabolic conditions such as hyperlipidemic states, obesity, metabolic syndrome, and diabetes and whether it has implications for metabolic health.

### Locations of TG Storage in the Gut

The exact locations of TG storage in the gut remain to be identified; however, several candidate locales appear to host the mobilizable pools. CLDs are one obvious source since they are a transient TG storage pool that undergoes dynamic metabolism during the feeding–fasting cycle (Hung et al., 2017). CLDs quickly expand in number and size following fat ingestion, while during the post-absorptive period, CLDs are catabolized, leading to fewer and smaller CLDs. The exact time frame of CLD synthesis and catabolism in humans has not been fully defined, but as mentioned above, a significant number and size of CLDs are still present many hours after fat ingestion (Robertson et al., 2003; Xiao et al., 2018b). Mature CM particles exit the enterocytes at the basolateral membrane by exocytosis into intercellular spaces and the lamina propria, before entering the lacteals, larger mesenteric lymphatics, and the thoracic duct, and ultimately enter the blood circulation. The presence of CM particles along this secretory pathway has been identified in the intercellular spaces, the lamina propria, and lymphatic vessels (Robertson et al., 2003; Takahara et al., 2013; Xiao et al., 2018b). These extracellular CMs, which appear to be retained for many hours after food ingestion (Xiao et al., 2018b), may also be a source of mobilized intestinal lipid and CMs in the post-absorptive period. Although not specifically demonstrated, we postulate that CMs within mesenteric lymphatics are also not all rapidly secreted into the blood circulation, likely represent another significant pool of ingested lipid that is retained for many hours following food ingestion, and could be mobilized by various stimuli of lymphatic pumping.

### Lipid Mobilization

Intestinal lipid pools are releasable upon receiving certain cues. In earlier studies, ingestion of a fat-rich meal hours after an earlier fat-rich meal that had elicited a typical postprandial TG excursion caused a rise in plasma TG prior to digestion and absorption of the second meal (Evans et al., 1998). In fact, sham fat feeding, i.e., chewing without swallowing of fats, also promoted appearance in CMs of TGs that originated from a previous meal (Mattes, 2009; Chavez-Jauregui et al., 2010). These studies point to a regulatory mechanism of gut lipid mobilization via neural circuits involving lipid sensing in the gut or through the olfactory and taste receptors, eliciting a “cephalic phase response” of CM secretion. Cephalic phase responses have been described for the secretion of hormones, such as insulin (Ahrén and Holst, 2001), ghrelin, and pancreatic polypeptide (Robertson et al., 2002; Simonian et al., 2005; Veedfald et al., 2016) prior to food digestion and absorption, triggered by sensing or perception of food. Cephalic phase secretion of hormones with sham feeding involves vagal efferent stimulation (Simonian et al., 2005), and both cholinergic and non-cholinergic autonomic activations mediate cephalic phase response of insulin secretion (Ahrén and Holst, 2001). Similarly, cephalic phase responses of intestinal lipid mobilization may involve vagal stimulation and increased parasympathetic activity (Robertson et al., 2002).

### Oral Glucose and GLP-2 as Stimuli of Intestinal Lipid Mobilization

Ingestion of glucose has been shown to promote gut lipid release. In healthy individuals, drinking a glucose solution 5 h after a high-fat liquid meal elicited rises in plasma and CM TG (Robertson et al., 2003; Xiao et al., 2018b). This was accompanied by depletion of lipid droplets in the enterocytes in the jejunum (Robertson et al., 2003) and shifting toward fewer and smaller CLDs in the duodenum (Xiao et al., 2018b). The underlying mechanism remains to be elucidated, but proteomic analysis of duodenal biopsies suggests differential regulation of several proteins, e.g., syntaxin-binding protein 5 and ethanolaminephosphotransferase, that may be components of the CM biosynthesis and assembly machinery (Xiao et al., 2018b). It is postulated that oral glucose, through yet to be defined mechanisms, mobilized CLD TGs for cycling back to the ER to be utilized as a substrate for CM synthesis, i.e., glucose ingestion triggered the recruitment of the cytosolic transient TG storage pool for CM synthesis and secretion.

Glucagon-like peptide-2 (GLP-2) is a potent stimulus of intestinal lipid mobilization. GLP-2 is a peptide hormone secreted by the intestinal L-cells in response to nutrient ingestion. GLP-2 infusion during meal ingestion potentiated postprandial TG excursion in humans (Meier et al., 2006). In animals, GLP-2 promoted CM secretion when administered with a fat load (Hsieh et al., 2009). More importantly, GLP-2 administered 5 h after a fat load also promoted CM secretion, suggesting mobilization of intestinal lipid stores (Hsieh et al., 2015). In two recent studies in healthy humans, we have examined the effects and regulatory mechanisms whereby GLP-2 affects lipid handling in the gut. We first demonstrated that GLP-2 rapidly and robustly elevated TGs in plasma and CM, which was mostly due to the release of “preformed” CM particles rather than *de novo* synthesis of CM (Dash et al., 2014). GLP-2 receptor (GLP-2R) expression pattern suggests that GLP-2 targets post-enterocyte lipid pools. The GLP-2R is not expressed on enterocytes (El-Jamal et al., 2014; Pedersen et al., 2015) that are responsible for CM intracellular synthesis and assembly. Instead, it is mostly identified on enteric neurons, myofibroblasts, and stromal cells (Yusta et al., 2000, 2019; Ørskov et al., 2005; Guan et al., 2006; Wismann et al., 2017), which are abundantly present in the subepithelial regions of the intestine, including the lamina propria. It has been suggested that GLP-2-induced intestinal wound repair may involve vascular endothelial growth factor (VEGF) secretion from fibroblasts (Bulut et al., 2008). GLP-2R is also expressed in cells positive for nitric oxide synthase (NOS) and vasoactive intestinal peptide, both with roles in regulating vessel dilation and blood flow (Guan et al., 2006). GLP-2 has been shown to stimulate mesenteric blood flow in humans (Bremholm et al., 2010, 2011; Høyerup et al., 2013) and animals (Guan et al., 2003, 2006; Deniz et al., 2007), which may involve nitric oxide (NO) signaling (Guan et al., 2003; Hsieh et al., 2015). Recently, we have examined lipid mobilization by GLP-2 in healthy humans with or without a NOS inhibitor L-NG-monomethyl arginine acetate (L-NMMA) (Xiao et al., 2019b). GLP-2 administration after 7 h of fat ingestion increased TG in plasma, in triglyceride-rich lipoprotein (TRL), and mostly in CM, and increased blood flow in the superior mesenteric artery. L-NMMA co-administration attenuated the effect of GLP-2 on the increase in blood flow, but without a noticeable effect on lipid mobilization. These results suggest that systemic NO does not play a prominent role in mediating GLP-2 mobilization of intestinal lipid stores. In a recent study in mesenteric lymph duct cannulated rats, we also demonstrate that glucose and GLP-2 mobilize intestinal lipid stores via distinct mechanisms, pointing to primarily intracellular and extracellular lipid pools by glucose and GLP-2, respectively (Stahel et al., 2019). Considering the anatomy of the intestinal region, the likely post-enterocyte locations for harboring lipid stores include intercellular spaces, lamina propria, and the mesenteric lymphatic vasculature.

## Lymphatics in the Regulation of Lipid Mobilization

### Intestinal Lymphatic System

The lymphatic system plays an important role in body fluid homeostasis, dietary fat absorption, inflammation and immune responses, and reverse cholesterol transport (Martel et al., 2013; Randolph and Miller, 2014; Bernier-Latmani and Petrova, 2017). Defects in the lymphatic system have been associated with metabolic disorders, including metabolic syndrome, obesity, diabetes, and atherosclerosis in animal models. Lymphatic function was impaired in rats with metabolic syndrome (Zawieja et al., 2012; Gasheva et al., 2019) and in obese mice (Weitman et al., 2013; Blum et al., 2014; García Nores et al., 2016), and defective lymphatics contributed to obesity and metabolism syndrome (Escobedo and Oliver, 2017; Gasheva et al., 2019). Diabetes in mice was associated with impaired lymphangiogenesis and disrupted lymphatic integrity (Scallan et al., 2015; Wu et al., 2018). The lymphatic system has also been implicated in the pathogenesis of atherosclerosis and cardiovascular disease (Aspelund et al., 2016). Impaired lymphatic function was associated with increased atherosclerosis, while enhancing lymphatic function attenuated development of atherosclerosis in mice (Vuorio et al., 2014; Milasan et al., 2019). Compromised lymphatic function has also been proposed to have systemic consequences for lipid metabolism and transport (Dixon, 2010).

Embryonic development of the lymphatic vessel growth and patterning and postnatal maintenance are under molecular regulation. Recent studies highlight critical roles of VEGF signaling in lymphangiogenesis during development and pathological processes (Alitalo, 2011). VEGF-C, the major lymphangiogenic factor, mediates lymphatic development and functions primarily through binding to VEGF receptor 3 (VEGFR3). In the intestine, VEGF-C is expressed by a subset of smooth muscle cells (SMCs) adjacent to the lacteals in the villus, in the circular smooth muscle layer of the intestinal wall, and in the arterial SMCs (Nurmi et al., 2015). In addition, VEGF-C is also secreted by villus macrophages upon recognition of gut microbes and their products (Suh et al., 2019). VEGF-C is activated by proteolytic cleavage of the full-length protein, a process involving the protease A disintegrin and metalloproteinase with thrombospondin motifs 3 and the secreted factor collagen and calcium binding EGF domains 1 (Bui et al., 2016; Jha et al., 2017). Besides being essential for perinatal lymphangiogenesis in many organs, VEGF-C, through activation of VEGFR3, is required for adult lymphatic vessel maintenance only in the intestine; thus, *Vegfc* deletion in adult mice leads to atrophy of intestinal lymphatic vasculature and lipid malabsorption (Nurmi et al., 2015). A distinct feature of intestinal lacteals is that they undergo continuous remodeling and are in a permanent regenerative and proliferative state in adults. This process is mediated by Notch signaling, and the expression of the Notch ligand delta-like 4 requires activation of VEGFR3 and VEGFR2 (Bernier-Latmani et al., 2015). Besides VEGF-C, adrenomedullin signaling through its receptor complex, calcitonin receptor-like (CRL) receptor, is also an important mediator for lymphangiogenesis during development and maintenance in adult mice (Hoopes et al., 2012; Davis et al., 2017). Lymphatic *Calcrl* (encoding CRL) deletion in adult mice compromises systemic lymphatic function, induces inflammation and lymphatic dilation in the gut, reduces lacteal proliferation, and impairs lipid absorption (Davis et al., 2017). This may be due to downregulation of the Notch signaling pathway that is essential for lacteal regeneration and function.

Collectively, development and maintenance of the intestinal lymphatic vasculature and function have important implications for lipid absorption in the gut (Cifarelli and Eichmann, 2019). We speculate, therefore, that regulation of intestinal lymphatic function plays an active role in the physiological control of the rate of dietary lipid secretion into the circulation, although experimental evidence is currently lacking.

### CM Entry Into the Lymphatics

The mesenteric lymphatic vasculature forms a network of lipid drainage. Following exocytosis, CM particles enter the intercellular spaces and the lamina propria. CMs are believed to move through the lamina propria via diffusion, largely influenced by convective movement of fluids (Tso and Balint, 1986). Subsequently, CMs enter the lymphatic system from the lamina propria via the lacteals (Takahara et al., 2013). Although a transcellular pathway has been described, the majority of CMs enter lacteals by a paracellular pathway via large, porous, intercellular junctions at the tip of the lacteal. Entry of CMs into the lacteal is believed to be through size exclusion; thus, the majority of CMs with considerable size enter the lacteal while only a minor fraction of CMs with very small sizes may enter the subepithelial blood capillaries (Takahara et al., 2013). Recent evidence in mice suggests that mechanisms beyond simple size exclusion may underlie the uptake of CMs by the lacteals. First, in mice lacking the transcription factor *PlagL2*, CMs can exit the enterocytes, but cannot enter the lacteals, resulting in accumulation of CMs in the lamina propria, fat malabsorption, and neonatal death (Van Dyck et al., 2007). A small fraction of CMs that do appear in the circulation are not efficiently utilized by peripheral tissues. The exact mechanism for this is not fully understood. *PlagL2* is expressed in the enterocytes and its deficiency reduces the expression of several genes (e.g., sorting nexins and vacuolar sorting proteins) that are candidate regulators of intracellular processing of dietary fat. It seems that particular CM particle properties, such as lipid and protein composition, are required for their successful entry into the lacteals, subsequent metabolism in the circulation, and utilization in peripheral tissues. Second, permeability of intercellular junctions on the lacteal wall is subject to regulatory control. Molecular control of the transition of vascular endothelial-cadherin (VE-cadherin) junctions between zipper (closed) and button (open) states impacts CM uptake into lacteals. Zippering of VE-cadherin junctions through VEGF-A-VEGFR2 signaling and VE-cadherin signaling resulted in CM malabsorption, which was rescued by restoring to buttoning state (Zhang et al., 2018). Third, VEGFR3, the major receptor that VEGF-C binds to, may play a role in regulating intestinal lipid absorption and CM entry into the lacteals. In the Chy mice that carry an inactivating mutation of the tyrosine kinase domain of VEGFR3, there was TG retention in the enterocytes, decreased postprandial plasma TG levels, and increased fecal excretion of free fatty acids and TGs, which may be mediated through NO in the intestine (Shew et al., 2018). Together, these studies support that passage of CMs through the lacteal endothelial wall involves mechanisms beyond simple size exclusion, requiring specific CM properties, controlled gating of the junctions, and VEGFR3 signaling.

### Lymphatic Pumping Functions and Regulation of CM Transport

Flow of lymph in the lymphatic vessel is achieved in part through an active pumping mechanism (Hosoyamada and Sakai, 2005; Kassis et al., 2016; Scallan et al., 2016). The smooth muscles surrounding the lymphatic endothelial wall contract in both tonic (vessel diameter) and phasic (frequency and amplitude) modes. The one-way valves inside the lymphatic vessel prevent lymph backflow. Together, contractile activities and valves provide unidirectional pumping force for active flow of lymph from the intestine to the circulation. Many of the above-mentioned compromised conditions, e.g., metabolic syndrome and obesity, are associated with impaired lymphatic functions, as reflected by reduced lymph drainage and decreased inflammatory cell mobilization and bacterial antigen clearance. For instance, the intrinsic contractility of the mesenteric lymphatics was impaired in a rat model of metabolic syndrome (Zawieja et al., 2012). Further, genetic manipulation to induce zippering (closing) of junctions along the lacteal wall impaired lacteal uptake of CMs and protected against diet-induced obesity in mice (Zhang et al., 2018). Taking advantage of the VEGF signaling in mediating lymphatic growth and function, chronic VEGF-C treatment has been applied to enhance lymphangiogenesis and lymphatic function, with enhanced lymphatic drainage, increased inflammatory cell mobilization, and bacterial antigen clearance in a mouse model of inflammatory bowel disease (D’Alessio et al., 2014).

While most studies demonstrate the importance of pre- and postnatal lymphangiogenesis in normal lymphatic vasculature development and maintenance of functions, studies also point to acute regulation of lymphatic functions. Lipid infusion into the duodenum of rats increased total lymph flow in the mesenteric lymphatic vessels, but decreased both phasic and tonic contractility (Kassis et al., 2016). Besides the major mesenteric lymphatics, intestinal lipid drainage in mice is regulated via contraction of the smooth muscles surrounding each lacteal, a process subject to the control of the autonomic nervous system (Choe et al., 2015). In rats, *in situ* perfusion of the mesenteric lymphatic bed with various VEGF signaling modulating factors acutely affected lymphatic pump activity (Breslin et al., 2007). Both VEGF-C and the VEGFR3-specific activator VEGF-C156S significantly increased contraction frequency, end-diastolic diameter, stroke volume index, and pump flow index. Conversely, inhibition of VEGFR3 caused tonic constriction and decreased contraction frequency and attenuated VEGF-C- and VEGF-C156S-induced lymphatic pump activation. This study supports that VEGFR3 mediates lymphatic pumping in the acute setting. Mechanisms other than VEGF signaling may also play a role in acute modulation of lymphatic functions. Contraction of isolated rat mesenteric lymphatics is reduced with the activation of the σ1-receptor through an NO-dependent mechanism (Trujillo et al., 2017). SMCs surrounding the intestinal lacteals express β-adrenergic receptors and muscarinic receptors (Bachmann et al., 2019), supporting a mediating role of the vagal nerves that provides dense innervation for intestinal lymphatics and surrounding muscles. Collectively, pumping functions of the larger mesenteric lymphatics and contraction of the lacteals actively participate in lipid drainage in the gut. Acute modulation of the functions of mesenteric lymphatics, including the lacteal, has been documented (Breslin et al., 2007; Choe et al., 2015). New and improved imaging techniques are being developed to specifically assess mesenteric lymphatic functions in relation to lipid drainage (Kassis et al., 2012; Sarimollaoglu et al., 2018). However, challenges remain in this aspect due to technical difficulties for such measurements (Bouta et al., 2018). It is hoped that with further improvement in techniques, the physiological relationship between lymphatic function and intestinal lipid drainage will be better defined.

## Future Directions

**FIGURE 1:**
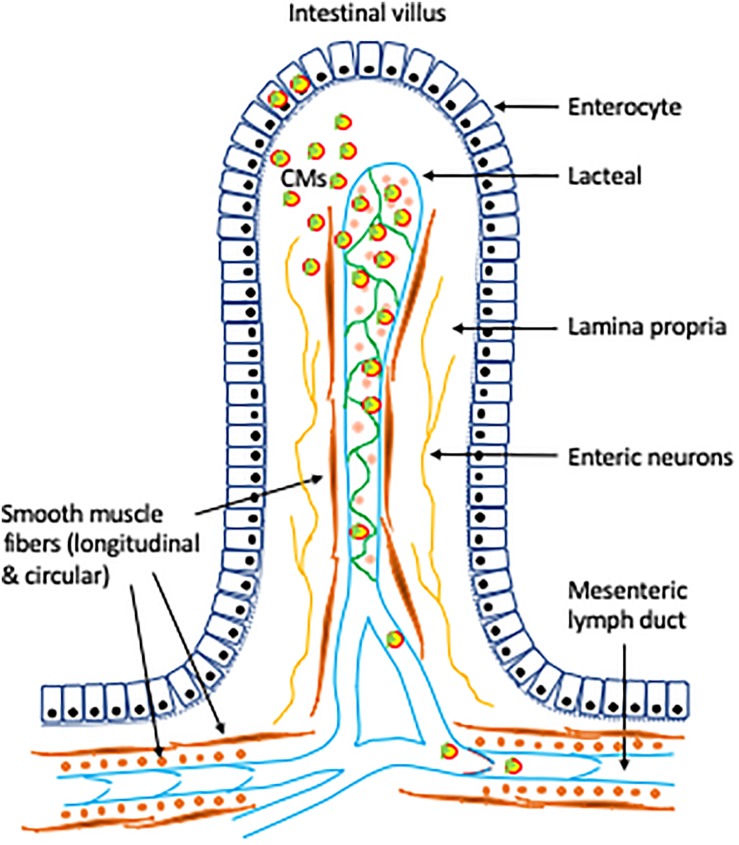
Role of lymphatics in intestinal lipid absorption and mobilization. Following intracellular synthesis and assembly in the intestinal enterocytes, CMs are secreted at the basolateral membrane via exocytosis. CMs move through the lamina propria and enter the lacteals through intercellular junctions. Controlled opening/closing of junctions on the lacteal wall may regulate CM uptake by the lacteals (Zhang et al., 2018). Contractile actions of smooth muscle fibers surrounding the lacteals and the collecting lymphatic vessels confer tonic and phasic pumping activities (Choe et al., 2015; Kassis et al., 2016). Lymphatic pumping and the one-way valves provide unidirectional drainage and transport of lipids through the lymphatic vasculature to the circulation. VEGF-C, expressed in a subset of smooth muscle fibers, plays an important role in intestinal lipid absorption and mobilization via regulation of prenatal lymphangiogenesis, maintenance of intestinal lymphatics in adults (Nurmi et al., 2015), and contraction of smooth muscle fibers surrounding the lymphatic vessels, including the lacteals (Gogineni et al., 2013; Choe et al., 2015). Neural control and σ1-receptor may also play a role in lymphatic functions (Trujillo et al., 2017; Bachmann et al., 2019).

Our research and that of others over the past 5 years have refocused our attention on post-enterocyte regulatory mechanisms of CM secretion. It is becoming evident that the lymphatics are likely to play a much more important regulatory role in this aspect than has traditionally been appreciated. Understanding the role of the lymphatics in lipid mobilization in the gut, as induced by oral glucose or GLP-2 and other stimuli, and in gut lipid handling in general, has important implications for health and disease. The mesenteric lymphatic system has emerged as a potential player in the regulatory control of intestinal TG absorption and mobilization (Figure 1). Despite recent advances in the field, many important questions remain to be answered. It is not known whether increased lymphatic pumping activity or induced button transition of lacteal junctions stimulates mobilization of intestinal lipid stores, or what the contribution these have to net CM secretory rate. It also remains to be determined whether increased lymphatic function underlies GLP-2 mobilization of intestinal lipid stores, and if it does, through what mechanisms. Further, it is not known whether lymphatic functions can be modulated, pharmacologically or via dietary means, to improve gut handling of lipids and provide overall health benefits. Future studies addressing these emerging and pressing questions may significantly improve our understanding of the physiological role of lymphatics in gut lipid metabolism. It is expected that advancement in this field may provide novel opportunities for the prevention and treatment of lipid disorders, atherosclerotic cardiovascular disease, and overall metabolic health.

## Author Contributions

CX and GL wrote the manuscript. PS and AN contributed and edited the manuscript.

## Conflict of Interest

The authors declare that the research was conducted in the absence of any commercial or financial relationships that could be construed as a potential conflict of interest.
